# *Petiveria alliacea *extracts uses multiple mechanisms to inhibit growth of human and mouse tumoral cells

**DOI:** 10.1186/1472-6882-8-60

**Published:** 2008-11-18

**Authors:** Claudia Urueña, Claudia Cifuentes, Diana Castañeda, Amparo Arango, Punit Kaur, Alexzander Asea, Susana Fiorentino

**Affiliations:** 1 Grupo de Inmunobiología y Biología Celular, Facultad de Ciencias, Universidad Javeriana, Bogotá, Colombia; 2 Division of Investigative Pathology, Scott & White Memorial Hospital and Clinic, Temple, Texas, USA; 3 The Texas A&M Health Science Center College of Medicine, Temple, Texas, USA

## Abstract

**Background:**

There is ethnopharmacological evidence that *Petiveria alliacea *can have antitumor activity; however, the mechanism of its cytotoxic activity is not well understood. We assessed multiple *in vitro *biological activities of an ethyl acetate soluble plant fraction over several tumor cell lines.

**Methods:**

Tumor cell lines were evaluated using the following tests: trypan blue exclusion test, MTT [3-(4,5-dimethylthiazol-2-yl)-2,5-diphenyl tetrazolium bromide], flow cytometry, cytoskeleton organization analysis, cell cycle, mitochondria membrane depolarization, clonogenicity test, DNA fragmentation test and differential protein expression by HPLC-Chip/MS analysis. F4 fraction characterization was made by HPLC-MS.

**Results:**

*Petiveria alliacea *fraction characterized by de-replication was found to alter actin cytoskeleton organization, induce G2 cell cycle arrest and cause apoptotic cell death in a mitochondria independent way. In addition, we found down regulation of cytoskeleton, chaperone, signal transduction proteins, and proteins involved in metabolic pathways. Finally up regulation of proteins involved in translation and intracellular degradation was also observed.

**Conclusion:**

The results of this study indicate that *Petiveria alliacea *exerts multiple biological activities *in vitro *consistent with cytotoxicity. Further studies in animal models are needed but *Petiveria alliacea *appears to be a good candidate to be used as an antitumor agent.

## Background

Inherent or acquired resistance can occur simultaneously to multiple drugs in the majority of tumor cells [1-4]. Almost 40% of cancer patients with resectable and 80% with unresectable disease have a reduced response to chemotherapy and radiotherapy. Several mechanisms have been associated with this resistance [5] and in order to overcome it, search for new antitumor agents must target different cell components within the tumor cell. In fact, single antitumoral compounds may be ineffective because of their unique molecular target. Therefore, presence of multiple compounds in well characterized plant extract with synergic activities, may tackle this difficulty since agonist or additive functions may emerge.

*Petiveria alliacea *L. (Phytolaccaceae) is a perennial shrub indigenous to the Amazon Rainforest, although it can grow in areas as Tropical and Central America, Caribbean and Southeastern United States. In folk medicine, *Petiveria alliacea*, is used to treat a wide variety of disorders. Root in decoction, powder or leaves infusion are used as antispasmodic, antirheumatic (topical use), anti-inflammatory [6,7], antinociceptive [8], hypoglycemiant and abortifacient [9,10]. Also there are reports describing the plant with sudorific, anti-venereal, diuretic, sedative, antihelminthic, emmenagogue, anesthetic and depurative [6,9] properties. In some South American countries, alcohol and water infusions have been used in patients with leukemia and breast cancer having good efficacy and reasonable toxicity at higher doses than commonly used by folk medicine [11-13].

Compounds isolated and reported for *Petiveria alliacea *includes flavonoids as astilbin, myricitrin, engeletin, triterpenes as barbinervic acid, α-friedelinol, steroids as daucosterol, lipids as lignoceric acid, nonadecanoic acid, oleic acid, compounds as allantoin, coumarin, [14-16], and several sulfur-containing amino acids in the roots; as well as S-benzylcysteine sulfoxides, and S-(2-hydroxyethyl) cysteine sulfoxides [17,18]. It is likely that benzylcysteine sulfoxides serve as precursors to thiosulfinates as S-(2-hydroxyethyl)-phenylmethanethiosulfinate and sulfines as thiobenzaldehyde S-oxide. Isolation and identification of three glutamyl dipeptides from roots of this plant have also been reported [19]. Dibenzyl trisulphide (DTS), a lipophilic compound found in the plant and identified as one of the immunomodulatory compounds [20], exhibiting anti-proliferative and cytotoxic activity were the cytoskeleton is implicated [21].

Several reports describe phytochemical characterization of *Petiveria alliacea's *ethanol and aqueous extracts, [11,14,16,17,22], and ethnopharmacological evidence describing possible antitumor activity [11]. This learning has not been immersed into common medical practice because lack of reliable experimental data. The present study examines cytotoxic activity *in vitro *of a partially purified *Petiveria alliacea *fraction over several tumor cell lines. Results warrant to continue toxicological and pharmacological testing that could lead to a role in tumor treatment.

For decades, pharmacognostic and ethnobotanical studies have focused in the search of single plant drug isolation, assuming that one drug is responsible for all plant biological activity. However, western medicine and even ayurveda, considers the possibility of synergy between different components in phytomedicine. Furthermore, there are clear examples where a single isolated compound is unable to reproduce the plant extract activity [23].

Current technical development in "omics" technology has permitted development of gene expression signatures for plant specific fractions. The latter technical advance allows validation of traditional plant uses, but unfortunately due to the high costs turns to be a technology quite inaccessible for developing countries. The present study, in addition to partial characterization of the plant fraction, we evaluate "protein expression signature" over melanoma tumor cells [24].

## Methods

### *Petiveria alliacea *fraction preparation

Plant material was collected in Viota, Cundinamarca, Colombia, and identified by Antonio Luis Mejia (botanical consultant) as *Petiveria alliacea *Linne. Plant material was compared with the Herbario Nacional Colombiano sample, registry number 333406 of August 12 de 1991. Dry ground leaves and stems (300 g) from *Petiveria alliacea *were extracted under reflux (60°C) with 1.5 liter of 96% ethanol for 3 h. The ethanol extract was filtered and evaporated until half its volume. An equal volume of water was added and heated (65°C) for 20 minutes to allow flocculation. The precipitate was eliminated by filtration and the liquid part subjected to liquid-liquid extraction with ethyl acetate (EtOAc) seven times. All the EtOAc fractions were combined and taken to dryness at 40°C under vacuum conditions. The dry extract was submitted to column chromatography on RP-C18 column (30 × 4 cm), and mobile phase methanol: water (MeOH:H_2_O). For ratio (1:1), 600 ml were eluted, yielding F-1 to F3 fractions. F-4 fraction eluted within the first 150 ml of ratio (7:3), and F-5 to F11 fractions eluted from the last 450 ml of ratio (7:3) and (9:1). F-1 to F11 fractions were assayed at concentrations ranging from 125 to 1.9 μg/ml but only fraction named F4 exhibited high cytotoxicity causing relevant changes in tumor cell lines morphology, reason why the biological testing was carryout on F4 fraction.

### Cell lines and growth conditions

Mel-Rel was established as a melanoma cell line from tumors developed in REL transgenic mice (gift from Dr. Armell Prevost, Cohin Hospital, Paris, France). A375 are human melanoma cells, courtesy of the Instituto de Investigaciones de la Universidad del Rosario (Bogotá, Colombia) and K562 a human erythroleukemia cell line from ATCC. Cells were placed in RPMI-1640 supplemented medium (10% FBS, 2 mM L-glutamine, 100 U/ml penicillin, 100 μg/ml streptomycin, 0.01 M Hepes) and incubated under humidified environment at 37°C and 5% CO_2_. Adherent cells at 75% of confluence were detached (trypsin/EDTA), washed (PBS) and suspended in complete medium. Human peripheral blood mononuclear cells (PBMC) from healthy volunteers were separated by density gradient centrifugation (Ficoll-Hypaque, Amersham, Biosciences) and the human fibroblasts from gingival tissue of healthy volunteers. PBMC and human fibroblasts were suspended in RPMI-1640 supplemented medium (10% FBS, 2 mM L-glutamine, 100 U/ml penicillin, 100 μg/ml streptomycin, 0.01 M Hepes) and incubated under humidified environment at 37°C and 5% CO_2_.

### In vitro cytotoxicity (IC50) and normal cell assays

All tumor cell lines were incubated and treated with F4 fraction (125 to 1.9 μg/ml), ethanol (0.2%), as negative control and vincristine (0.1 to 0.0015 μg/ml) as positive control, during 48 h at 37°C. Adherent cells trypsinized, and washed with saline phosphate buffer (PBS). Human PBMC and fibroblasts were seeded (2 × 10^5 ^cells/well) on 96-well plates and incubated with or without phytohemagglutinin (PHA, GibcoBRL) for 12 h. Afterwards, PBMC and fibroblasts were treated with F4 fraction (125 to 1.9 μg/ml), ethanol (0.2%) and vincristine, for 60 h and 24 h, respectively. After treatment cells were centrifuged, F4 fraction removed and lastly cells were carefully washed 3 times (PBS) before adding the MTT. Next 12 μl of MTT 12 mM [3-(4,5-dimethylthiazol-2-yl)-2,5-diphenyl tetrazolium bromide] (Molecular Probes, Eugene, Oregon, USA) in PBS was added to each well and incubated for 4 h at 37°C. Formazan crystals were dissolved with SDS-HCl 0.01 M. MTT results were read at 540 nm in a Multiskan MCC/340 (LabSystems). In addition cell viability was assessed with a trypan blue dye exclusion test. The IC50 (50% inhibition of cell growth) value was calculated using Probit analysis (MINITAB^® ^Release 14.1. Minitab Inc. 2003 Statistical Software).

### Cell cycle analysis

Mel Rel, A375 and K562 tumor cells lines, starved for 72 h (to induce arrest in G1 phase), seeded in 12-well plate (4 × 10^5 ^cells/well) were treated with concentrations of F4 fraction at 12, 18, 24 and 48 h under humidified environment at 37°C and 5% CO_2_. After treatment, cells were washed and fixed with ethanol (70%, ice-cold) during 18 h. After fixing, cells were suspended in PBS 1X, 100 U/ml RNase, 50 μg/ml of propidium iodide (Sigma, St. Louis, MO) and incubated at room temperature for 30 min. Cell DNA content was measured by flow cytometry using a FACScalibur, (Becton Dickinson, Fullerton, CA). For cytometric data 50,000 cellular events were collected per sample and analyzed with Cell Quest software (Becton Dickinson). Cell cycle distribution percentages are calculated by Modfit LT software. FACScalibur calibration is performed with the DNA QC Particle Kit (Becton Dickinson). Treatments were performed in triplicate, and results express as mean ± SEM.

### Cytoskeleton organization analysis

A375 human cells (5 × 10^4 ^cells/ml) plated on glass coverslides (13 mm diameter), precoated with collagen (Sigma, St. Louis, MO) were allowed to adhere for 16 h. Afterwards, treated with F4 fraction for 24 h and incubated under humidified environment, at 37°C and 5% CO_2_. Treated cells were washed (PBS) and fixed (2% paraformaldehyde in PBS) for 30 min at 4°C. Fixed cells were wash twice with 1% PBS-BSA, incubated with cold acetone for 1 min, washed (1% PBS-BSA) and incubated with phalloidin conjugated to Oregon-green (Molecular Probes, Eugene, Oregon, USA), diluted in 1% PBS-BSA (1/40) for 30 min. Slides were mounted with prolong antifade kit (Molecular Probes, Eugene, Oregon, USA) and analyzed under fluorescence microscope (Olympus, Japan).

### DNA fragmentation analysis

A375 human cells were treated and incubated as described on cytoskeleton organization procedures except for last step were cells are stained with 300 nM of DAPI (Sigma, St. Louis, MO) for 5 min. Slides were mounted with prolong anti-fade kit (Molecular Probes, Eugene, Oregon, USA) and cells analyzed under fluorescence microscope (Olympus, Japan).

### Clonogenic assays

K562 human cells (2.5 × 10^5 ^cells/well) plated (96-well plate) were treated with F4 fraction at 31.2, 15.6 and 7.8 μg/ml, or 200 μg/ml etoposide, or 0.1 μg/ml vincristine or 0.2% ethanol (in PBS) and incubated for 24 h under humidified environment at 37°C and 5% CO_2_. After treatment cells were re-plated onto 0.5% agar dishes (60-mm, 20,000 cells/dish), incubated for 14 days (37°C and 5% CO_2_) and stained with violet crystal (0.4% in ethanol). Cell colonies with more than 50 cells were counted. Treatments were performed in triplicate, and results expressed as mean ± SEM.

### Evaluation of Mitochondrial Membrane potential (MMP)

Mitochondria membrane potential (MMP) was measured on human K562 cells by flow cytometry, using JC-1, a lipophilic cationic probe (5,5',6,6'-tetrachloro-1,1',3,3'-tetraethyl-benzimidazolcarbocyanine iodide), (Sigma, St. Louis, MO). JC-1 (10 μg/ml in PBS) is added to 3 × 10^5 ^cells/ml and incubated for 10 min at 37°C. Data analysis was processed by Cell Quest software (Becton Dickinson). All treatments were performed in triplicate, and results expressed as mean ± SEM.

### Characterization and identification of proteins

#### Sample Preparation

A375 cells treated with F4 fraction (31.2 μg/ml for 24 h) lysed in lysis buffer, supplemented with phosphatase and proteinase inhibitors. Protein samples were de-salted in 10 K microcon, diluted with 100 ml of ammonium bicarbonate buffer (100 mM). Cysteine residues were reduced with DTT (10 mM) by incubation at 65°C for 45 min. After cooling to room temperature, sulfhydryls were alkylated with iodoacetamide (55 mM) for 30 min at room temperature in a dark environment. The reduced and alkylated sample was diluted (1:1) with water. Trypsin (Promega, Madison, WI) was added at a 1:50 enzyme:substrate ratio, and incubated overnight at 37°C. Tryptic peptides were completely dried in a SpeedVac and reconstituted with 10 ml of 0.1% TFA.

#### HPLC-Chip/MS analysis

A 1 ml sample of peptides was injected onto an LC/MS system consisting of an 1100 Series liquid chromatograph, HPLC-Chip Cube MS interface, and 1100 Series LC/MSD Trap XCT Ultra ion trap mass spectrometer (all Agilent Technologies). The system is equipped with an HPLC-Chip (Agilent Technologies) that incorporated a 40-nl enrichment column and a 43-mm × 75-mm analytical column packed with Zorbax 300SB-C18 5-mm particles. Peptides were loaded onto the enrichment column with 97% solvent A (water with 0.1% formic acid). They were then eluted with a gradient from 3% B (acetonitrile with 0.1% formic acid) to 45% B in 25 min, followed by a steep gradient to 90% B in 5 min at a flow rate of 0.3 ml/min. The total runtime, including column reconditioning, was 35 min. The column effluent was directly coupled to an LC/MSD Trap XCT Ultra ion trap mass spectrometer (Agilent Technologies) via a HPLC-Chip Cube nanospray source operated at ~1900 volts in ultra-ultra mode. The gain control (ICC) was set to 500000 with a maximum accumulation time of 150 milliseconds. CID was triggered on the six most abundant, not singly charged peptide ions in the m/z range of 450–1500. Precursors were set in an exclusion list for 1 min after two MS/MS spectra.

#### Data analysis

CID data was searched against the SwissProt all species database, using the Agilent Spectrum Mill Server software (Rev A.03.03.) installed on a HP Intel^® ^Xeon (TM) dual processor server. Peak lists were created with the Spectrum Mill Data Extractor program with the following attributed: scans with the same precursor ± 1.4 m/z were merged within a time frame of ± 15 s. Precursor ions needed to have a minimum signal to noise value of 25. Charges up to a maximum of 7 were assigned to the precursor ion, and the 12C peak was determined by the Data Extractor. The SwissProt database was searched for tryptic peptides with a mass tolerance of ± 2.5 Da for the precursor ions and a tolerance of ± 0.7 Da for the fragment ions. Two missed cleavages were allowed. A Spectrum Mill auto-validation was first performed in the protein details, followed by peptide mode using default values [Minimum scores, minimum scored peak intensity (SPI), forward minus reversed score threshold, and rank 1 minus rank 2 score threshold]. All protein hits found in a distinct database search by Spectrum Mill were non-redundant. Analysis of the increase or decrease in proteins was performed by comparing each sample with the control. Those values above and below 0.250 from the control value were considered up- or down-regulated.

#### HPLC-PDA MALDI-TOF

HPLC chromatogram was recorded on a Waters HPLC Alliance 2690 (Waters, Milford, MA) chromatograph with PDA detector (Waters 2690), and RP-C18 column (5 μm, 2.1 × 150 mm, Waters), at 0.3 ml/min with acetonitrile-water (4:6). MALDI-TOF spectra was recorded in a mass spectrometer (Bruker Reflex III), equipped with a 337 nm N_2 _laser and HCCA matrix.

#### Statistical analysis

The mean fluorescent intensity was used to compare flow cytometry data of controls and samples and expressed as the mean ± SEM. The unpaired Student's *t*-test was used (p < 0.05) to measure differences between treatments and controls. IC50 was estimated using Minitab 14 Statistical Software Probit analysis [(MINITAB^® ^Release 14.1. Minitab Inc. 2003 Statistical Software).

## Results

### F4 fraction Characterization

**Figure 1:**
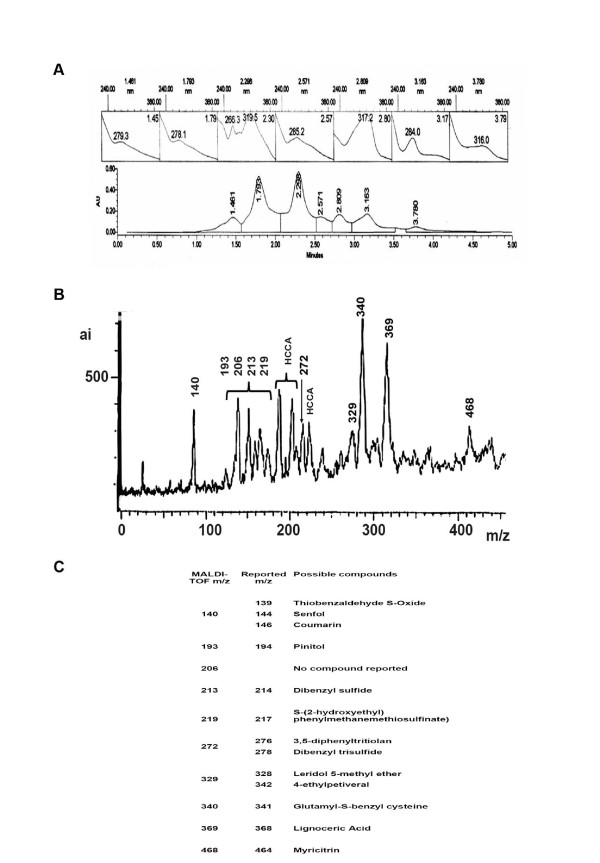
***Petiveria alliacea *F4 fraction characterization**. A. Upper panel shows compound UV spectra and retention time. Lower panel shows peak area and retention time. B. F4 fraction was subjected to MALDI-TOF-MS analysis. Numbers above the peaks correspond to m/z ratios. The horizontal axis represents the mean relative intensity and the abscissa m/z ratios. HCCA peaks correspond to matrix (4-cyano-4 hydroxy-cinnamic acid) signals. C. m/z ratio of compounds reported for *Petiveria *and compounds found in F4 fraction matching within (± 7) units of the m/z ratio.

Fig. 1A shows 7 peaks. Peak 2 and 3 accounts for approximately 60% of total area. Maximum absorption λ (lamda) for peak 2 is 278 nm, and for peak 3, 266 and 319 nm. Peak 6 accounts for 12% of the total area, with maximum absorption λ at 284 nm. Peaks 1, 4, 5 and 7 independently, exhibit lower percentages, but combined account for 27% of the total fraction. Peaks maximum absorption λ are 279, 285, 317 and 316 nm, respectively. Fig. 1B shows F4 fraction mass spectra profile including 10 peaks with the following mass/charge (m/z) ratios: 140, 193, 206, 213, 219, 272, 329, 340, 369, and 468. The HCCA peaks correspond to matrix (4-cyano-4 hydroxy-cinnamic acid) signals. Peaks with m/z of 340 and 369 have the higher concentrations, while peaks of m/z ratios 140, 193 and 206 reveal intermediate concentrations. Peaks with m/z ratios of 213, 219, 272, 329 and 468 have the lowest concentrations. Fig. 1C shows possible compounds present in F4 fraction with their corresponding molecular masses. Compound identification accomplish by matching the MALDI-TOF m/z with molecular weights of compounds previously reported for *Petiveria *[14-17,22]. The m/z ratio given by MALDI-TOF spectra comprises a deviation range of ± 7 mass units, due to the method used. This difference was taken into account for compound identification. The observed peak (m/z = 140) represents three possible compounds: thiobenzaldehyde-S-oxide, 1,2 diisothiocyanato ethane (senfol) and coumarin, with molecular masses of 139, 144 and 146 respectively. The peak of m/z = 193 probably corresponds to pinitol (194), and peak 206, no compounds matching that m/z ratio are reported for *Petiveria alliacea*. Peaks with m/z of 213, 219 and 272 respectively, correspond to sulfur compounds: dibenzyl sulfide, S-(2-hydroxyethyl)-phenylmethanethiosulfinate and for peak 272 two compounds; 3,5-diphenyltritiolan (276) and dibenzyltrisulfide (278). A m/z of 329 corresponds to flavonoid 5-O-methyl leridol, while a m/z of 340 has two possible compounds 4-ethyl petiveral and glutamyl-S-benzyl cysteine. Finally peaks 369 and 468 correspond to lignoceric acid and myricitrin respectively. Since compound identification was carry out comparing m/z ratio calculated by MALDI-TOF with the reported m/z ratios, identification of stereoisomers by this approach is not feasible. Based on m/z ratios, the compounds described for the F4 fraction are thought to be those presented. However, definitive identification is underway.

### *Petiveria alliacea *F4 fraction induces morphological changes on tumor cell lines without affecting normal human cells

**Figure 2:**
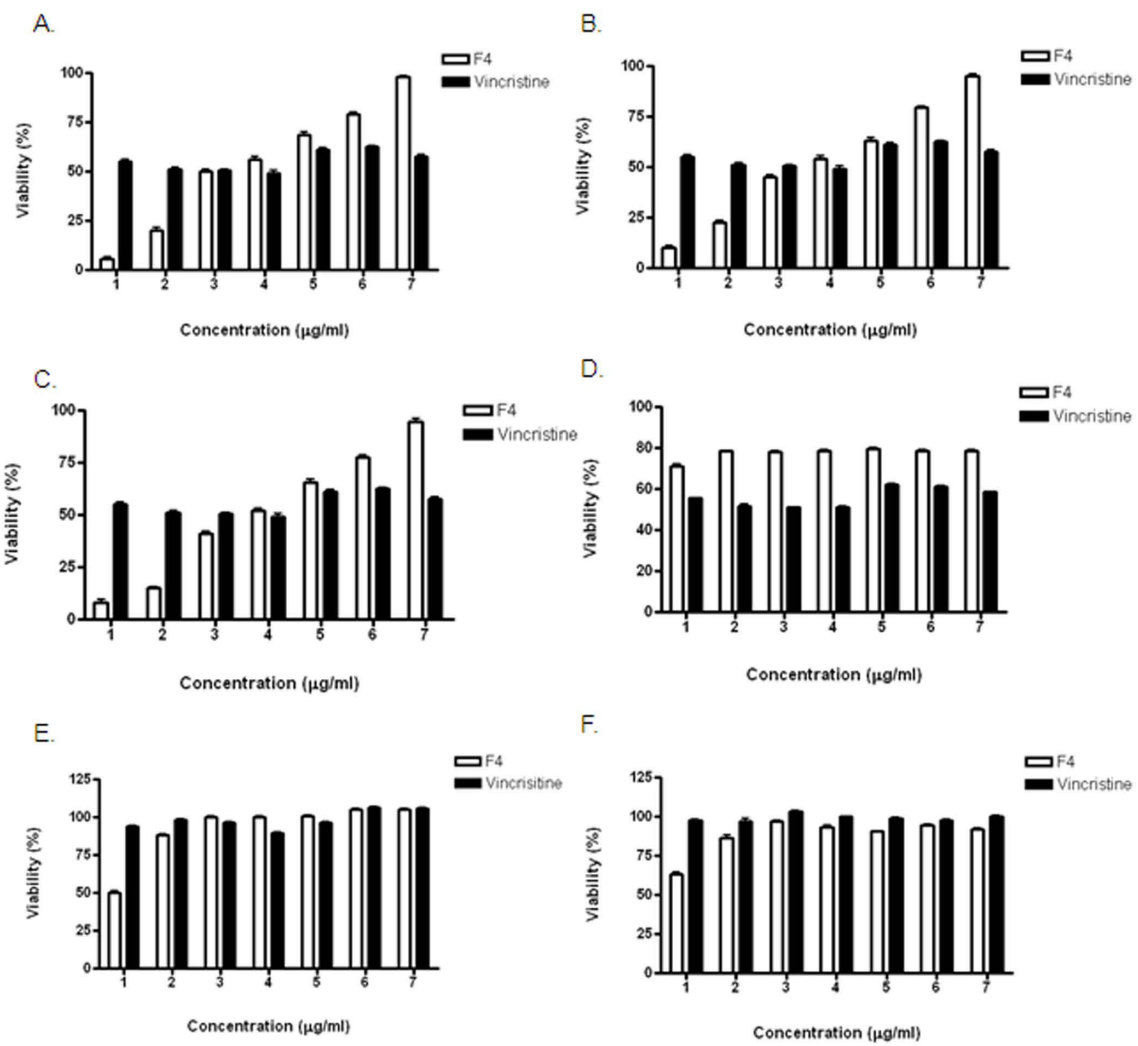
***Petiveria alliacea *F4 fraction is cytotoxic to tumor cell lines without affecting human normal cells**. A. A375 B. Mel Rel C. K562 D. Human fibroblasts E. PBMC stimulated with PHA or F. PBMC-PBS treated with F4 fraction concentrations (white) at 125 (1), 62.5 (2), 31.2 (3), 15.6 (4), 7.8 (5), 3.9 (6), and 1.8 μg/ml (7); or vincristine (black) 0.1 (1), 0.05 (2), 0.025 (3), 0.0125 (4) 0.00625 (5), 0.0031 (6) and 0.0015 μg/ml (7), for 24 h. Cell viability was determined by MTT assay as described in the methods section. Data represent cell viability percentage (%), where the vehicle-treated cells are regarded as 100%. The values are mean ± SEM from three independent experiments.

**Figure 3:**
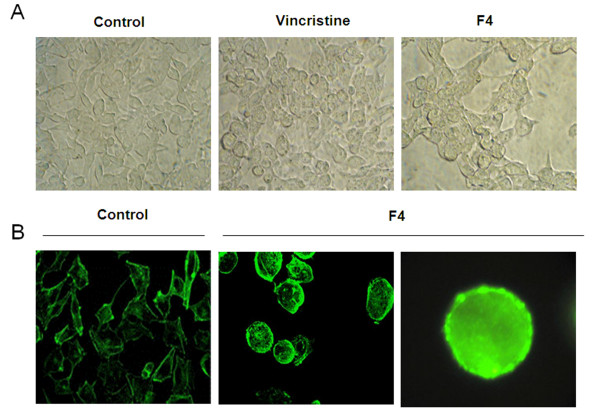
**F4 fraction induces morphological changes in tumor cells**. A. A375 cells treated with ethanol 0.2% (left panel), vincristine 0.1 μg/ml (middle panel) or F4 fraction 31.2 μg/ml (right panel). Morphological changes were analyzed under invert microscope. Results represent three independent performed experiments. B. A375 (10^4^) treated with ethanol 0.2% (left panel) or F4 fraction 31.2 μg/ml (middle and right panels) for 24 h. Cells were stained with Oregon Green-phalloidin were analyzed under fluorescent microscope. Results show photos representing four independent experiments.

F4 fraction cytotoxic activity evaluated by MTT assay is shown on figures 2A, B and 2C. The cytotoxicity of F4 fraction is dose dependent inducing significant morphological changes, as cell deformation and elongation in similar way to vincristine in all tested tumor cell lines (Fig. 3A). According to IC50, F4 fraction shows similar cytotoxic potency on A375, Mel Rel and K562 tumor cells lines with values of 35,2, 36,3 and 32,0 μg/ml respectively (Table 1). Comparing cytotoxic activity between tumor cell lines and normal human cells, F4 fraction exhibits significantly less cytotoxicity on normal fibroblasts (IC50 440 μg/ml) (Fig. 2D and Table 1) or human mononuclear cells with or without phytohemaglutinin (PHA) (IC50 151, 121 μg/ml respectively) (Fig. 2E, F and Table 1). F4 fraction is by far the most promising fraction owing significant difference in cytotoxicity for tumor cell lines as compared with normal cells, explaining the reason why F4 fraction was extensively studied.

**Table 1:** Comparative IC50 values of F4 fraction and vincristine over tumor cell lines and normal human cells.

**CELLS**	**A375**	**K562**	**Mel Rel**	**PBMC no PHA**	**PBMC with PHA**	**Fibroblasts**
**F4 fraction (IC50 μg/ml)**	35.2 ± 1.35*	32 ± 1.41	36.3 ± 1.64.	121 ± 2.6 *	151 ± 8.3 *	440 ± 15 *
**Vincristine (IC50 nM)**	132 ± 10*	61 ± 4*	124.5 ± 15*	247 ± 22*	197 ± 20*	85.5 ± 24*

± = SEM, p < 0.05

The IC50 values of tumor cell lines and normal human cells treated with F4 fraction were calculated with Minitab 14 Statistical Software Probit analysis. The values are mean ± SEM from three independent experiments.

In addition, we observed that tumor cells treated with F4 fraction underwent morphological changes in shape, adhesion ability and induced G2 phase arrest. To further study F4 fraction activity on actin cytoskeleton organization, cells treated with F4 fraction were stained with phalloidin-oregon green conjugate. As observed in Fig. 3B, actin cytoskeleton organization was disturbed after 24 hours. A375 cells treated with F4 fraction did not show the same fluorescence pattern as observed in control cells (ethanol 0.2%). Vehicle cells showed considerable F-actin cytoskeleton organization (Fig. 3B; left panel), while cells treated with F4 fraction showed differences in shape, displaying reorganized filamentous structures (Fig. 3B; middle and right panels). The latter indicates that actin filaments were transformed into actin granules confining at the cell sub-membrane area. Similar results were obtained with cell line Mel-Rel (data not shown).

### F4 fraction induces apoptosis in a mitochondria independent way

**Figure 4:**
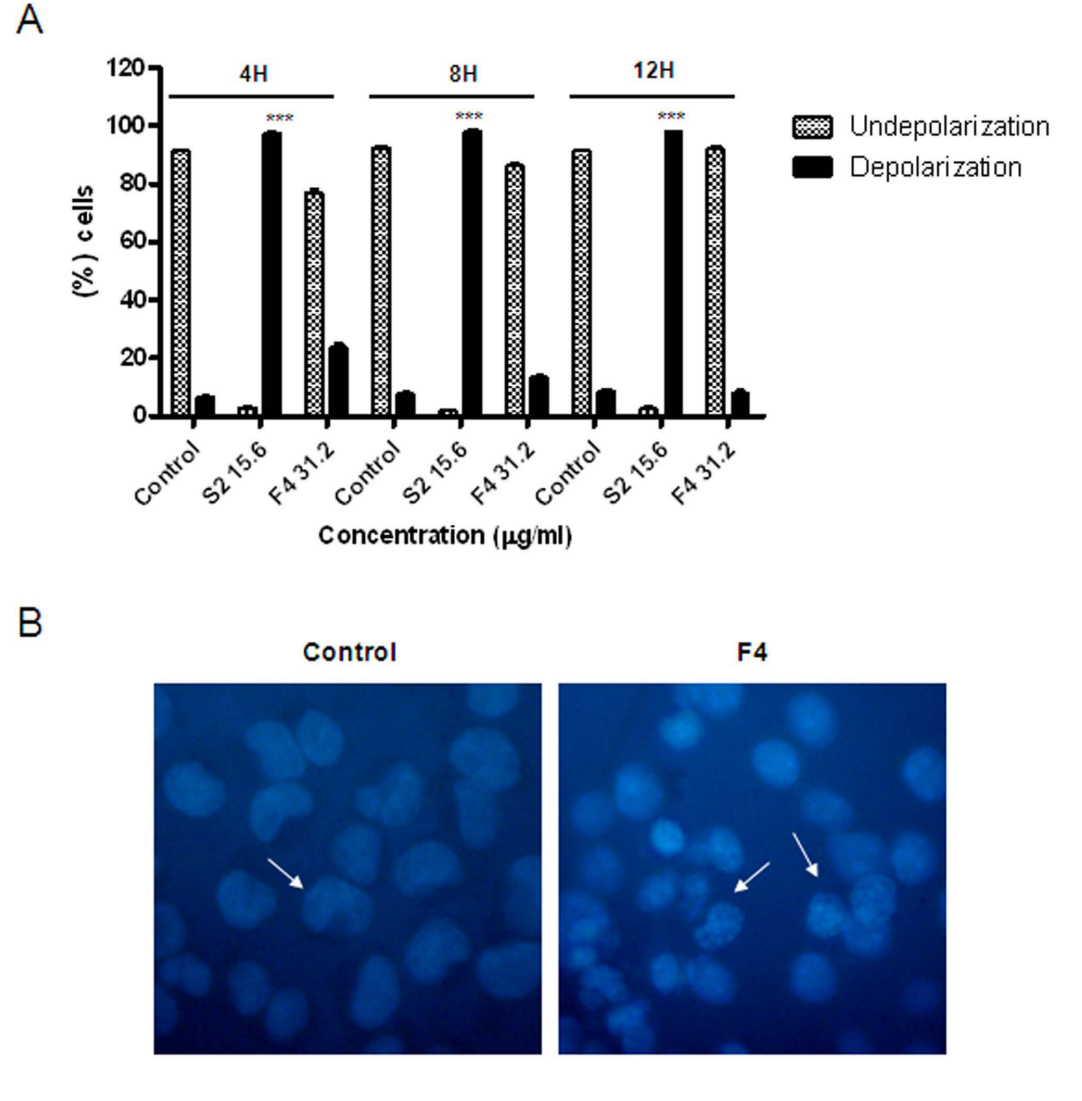
**F4 fraction has no activity on mitochondrial membrane depolarization**. A. K562 human cells were treated with F4 fraction (31.2 μg/ml), or positive control S2 fraction (15.6 μg/ml) or ethanol (0.2%) for 4, 8 and 12 h. All cells were stained with JC-1 (Sigma) dye and analyzed by flow cytometry (FACScalibur CellQuest software program) (Becton Dickinson). Bars represent cell percentage (%) ± SEM of depolarized (filled bars) or non-depolarized (hatched bars) cells, representing two independent experiments. ***p < 0.001 versus control (ethanol 0.2%; Unpaired Student's *t*-test). B. A375 cells treated with ethanol (0.2%) (left panel) or F4 fraction (31.2 μg/ml) (right panel) for 24 h were permeabilized, stained with DAPI and analyzed under fluorescence microscope (Olympus). Results show photos representing four independent experiments.

Antitumor drugs commonly induce apoptosis via mitochondria, liberating cytochrome c, activating endonucleases, and ending in DNA fragmentation. However, F4 fraction did not induce mitochondrial membrane depolarization in K562 cells, contrasting with the behavior shown by S2 fraction (positive control used), which is also a *Petiveria alliacea *fraction that induces mitochondrial depolarization (Fig. 4A). Nonetheless, F4 fraction instead stimulates endonuclease activation and DNA fragmentation shown by staining with DAPI and analyzed by fluorescence microscopy on A375 cells (Fig. 4B). This suggests that F4 fraction activates effector caspases in a mitochondria independent pathway.

### Effect of F4 fraction on tumor cell cycle distribution

**Figure 5:**
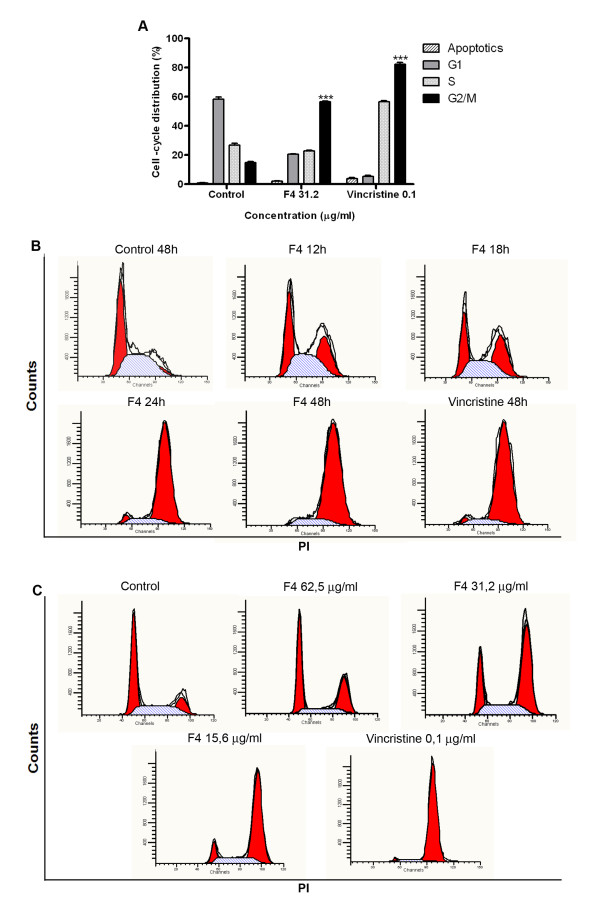
**Effect of *Petiveria alliacea *F4 fraction on cell cycle**. A. A375 cells treated with ethanol (0.2%), or F4 fraction (31.2 μg/ml) or vincristine (0.1 μg/ml) for 24 h, were permeabilized, stained with propidium iodide (PI) 50 μg/ml and analyzed through flow cytometry (FACScalibur CellQuest software program) (Becton Dickinson). Bars represent relative percentage of cell-cycle distribution ± SEM and represent three independent performed experiments. ***p < 0.001 versus control (ethanol 0.2%; Unpaired Student's *t*-test). B. A375 cells treated with ethanol (0.2%) or F4 fraction (31.2 μg/ml) or vincristine 0.1 μg/ml for 12, 18, 24 and 48 h, were permeabilized, stained with PI 50 μg/ml and analyzed through flow cytometry (FACScalibur CellQuest software program) (Becton Dickinson). Histograms represent relative cell DNA content representing two independent experiments. C. A375 cells treated with ethanol (0.2%) or F4 fraction(62.5, 31.2, 15.6 μg/ml) or vincristine (0.1 μg/ml) for 48 h, were permeabilized, stained with PI 50 μg/ml and analyzed through flow cytometry (FACScalibur CellQuest software program) (Becton Dickinson). Histograms represent relative cell DNA content representing two independent experiments.

To further study the effect of F4 fraction on tumor cell lines, cell cycle distribution was assessed on A375, K562, and Mel-Rel cell lines by flow cytometry, staining the DNA content with propidium iodide. Cells treated with a single dose of F4 fraction (31.2 μg/ml) induced G2 arrest (60%) as compared with a negative control (18%) ethanol (0.2%). Vincristine (0.1 μg/ml), positive control induced G2 arrest (80%) as shown on Fig. 5A. K562 and Mel Rel behave in the same manner (data not shown). In addition, F4 fraction activity over cell cycle kinetics was further investigated, by synchronizing A375 cells in a pulse chase experiment over 48 h. The G2 arrest in A375 cells lasts 48 h as shown in Fig. 5B. The G2 arrest was produce at 31.2 and 15.6 μg/ml but not at higher concentrations (Fig. 5C).

### F4 fraction reduces tumor cells clonogenic survival

**Figure 6:**
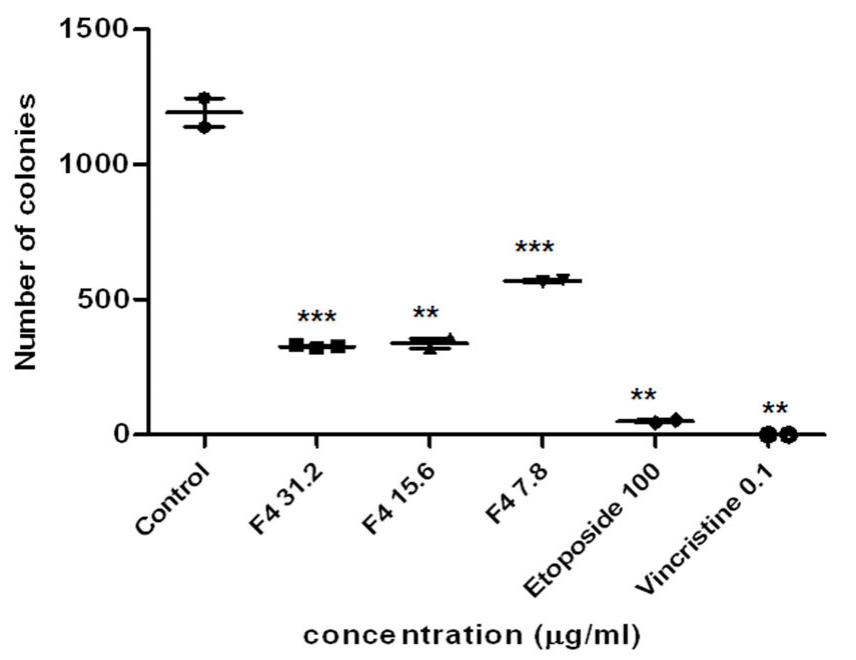
**F4 fraction abrogates K562 erythroleukemic cells colony forming ability**. K562 cells plated at 2.5 × 10^5 ^cells/well were treated with ethanol (0.2%), or F4 fraction (from 7.8 to 31.2 μg/ml), or etoposide (100 μg/ml), or vincristine (0.1 μg/ml) for 24 h. Afterwards, cells were stained with crystal violet (0.4% in ethanol). Data represents number of colonies ± SEM representing two independent experiments. ***p < 0.001 versus control (ethanol 0.2%) Unpaired Student's *t*-test).

K562 human cell line treated with F4 fraction significantly reduced cell colony formation, as compared with vehicle cells (0.2% ethanol). Colonies were evaluated after 14 days of treatment (Fig. 6) and a decrease in clonogenic survival was observed in treated cells as compared to a negative control (0.2% ethanol). However, the decrease in clonogenic survival exhibited by positive controls, etoposide (100 μg/ml) and vincristine (0.1 μg/ml) was slightly greater. Similar data was observed on A375 cells (data not shown).

### Proteomic characterization of F4 fraction activity over tumor cells

To better understand the mechanism by which F4 fraction exerts its cytotoxic activity on tumor cell lines, A375 (human) cells were treated with F4 fraction (15 and 31.2 μg/ml) or ethanol (0.2%) (negative control) for 24 hours. The protein content was analyzed by mass spectrometry. Experimental analysis demonstrated that various proteins were either up- or down-regulated (Table 2 and 3). Out of 201 proteins 76 were up-regulated, 114 were down-regulated and 11 remained unchanged. Ribosomal proteins (e.g., ribosomal protein L4, 5, 7a, 8, 9, 10, 10a, 11, 12, 13, 14, 18, 18a, 19, 23, 26, 27, 27a, 28, 32, 36, 37a, ribosomal protein S2, 4, 5, 6, 8, 11, 12, 13, 15a, 18, 19, 24, 25, 27, 31 and ribosomal protein P0 variant) are molecules important for tumor growth and survival. Cytoskeleton proteins (e.g., lamin B1, dynein light chain 1, plectin, t-complex polypeptide 1 (TCP-1), chaperonin containing TCP-1 (subunit 6 A and 7), kinesin, tubulin alpha 6, myosin heavy polypeptide 9, actin, gamma 1 propeptide, adenylyl cyclase-associated protein, F-actin capping protein alpha 1, Miller-Dieker lissencephaly protein, myosin, light polypeptide 6B, alkali, smooth muscle and non-muscle, (isoform CRA) are also down-regulated.

**Table 2:** Proteins down regulated by F4 Fraction

**Down regulated proteins**	**Accesion Number**
**Translation**	
Asparaginyl-tRNA synthetase	NP_004530
BAT1 protein	BAF31287
CGI-74 protein	AAD34069
Dhx9 (DEAH) P-9, RNA Helicasa A (RHA)/DEAH (Asp-Glu-Ala-His)	NP_001348
Eukariotic translation elongation factor 1 gamma	AAH13918
Eukariotic translation elongation factor-2 (EF-2).	NP_001952
Eukaryotic initiation factor 4A (eIF-4A)	NP_001407
Heparin-binding protein HBp15 solo un articulo	AAP97261
Heterogeneous nuclear ribonucleoprotein H1	NP_005511
Heterogeneous nuclear ribonucleoprotein U isoform a (scaffold attachment factor-A)	NP_114032
Interleukin enhancer binding factor 3, 90 Kda	EAW84137
Nucleolin	NP_005372
Nucleosome assembly protein 1-like 1	NP_631946
Poly(rC)-binding protein 2 isoform b variant PCBP2	NP_114336
Small nuclear ribonucleoprotein Sm D1	CAE11897
Tryptophanyl-tRNA synthetase (IFP53)	CAA44450
Tu-transcription elongation factor. ET-1 o EF-Tu	NP_003312
	
**Transcription processing**	
CGI-74	AAD34069
DEAD (Asp-Glu-Ala-His) box polypeptide 21	NP_004719
DEAD (Asp-Glu-Ala-His) box polypeptide 9	NP_001348
	
**Ribosomal Proteins**	
Ribosomal protein L4	NP_000959
Ribosomal protein L5	EAW73088
Ribosomal protein L7a	EAW88064
Ribosomal protein L8	NP_000964
Ribosomal protein L9	NP_000652
Ribosomal protein L10	NP_006004
Ribosomal protein L10a	NP_009035
Ribosomal protein L11	NP_000966
Ribosomal protein L12	BAD92708
Ribosomal protein L13	NP_150254
Ribosomal protein L13a	AAQ13495
Ribosomal protein L14	NP_004964
Ribosomal protein L18	NP_000970
Ribosomal protein L18a	XP_943475
Ribosomal protein L19	EAW60568
Ribosomal protein L23	NP_000969
Ribosomal protein L26	NP_001087230
Ribosomal protein L27	NP_000979
Ribosomal protein L27a	NP_001083056
Ribosomal protein L28	NP_000982
Ribosomal protein L31	NP_000984
Ribosomal protein L32	NP_000985
Ribosomal protein L36	NP_378669
Ribosomal protein L37a	NP_000989
Ribosomal protein S2	NP_002943
Ribosomal protein S4	EAW71815
Ribosomal protein S5	BAD93040
Ribosomal protein S6	NP_001001
Ribosomal protein S8	EAX07023
Ribosomal protein S11	NP_001006
Ribosomal protein S12	EAW54624
Ribosomal protein S13	NP_001008
Ribosomal protein S15a	EAW50259
Ribosomal protein S18	NP_001087248
Ribosomal protein S19	NP_001013
Ribosomal protein S24	EAW54624
Ribosomal protein S25	NP_001019
Ribosomal protein S27	EAW91426
Ribosomal protein P0 variant	BAD96291
	
**Replication **	
CSE1 chromosome segregation 1-like protein	NP_001307
ErbB3 (HER3) binding protein 1	NP_006182
Nucleolin	NP_005372
Protein Kinase, DNA-activated, catalytic polypeptide isoform 1	NP_008835
	
**Degradation Proteins**	
26S proteasome subunit p45	BAA07919
Flap structure-specific endonuclease 1	NP_004102
Fumarate hydratase, isoform CRA_b	EAW70091
Histone cluster 1, H1d	NP_005311
HMG-1	BAA09924
Hydroxysteroid (17-beta) dehydrogenase 10 isoform 1	NP_004484
Mitochondrial acetoacetyl-CoA thiolase	BAA01387
PSMC3 protein	AAI07805
	
**Transporters**	
Mitochondrial trifunctional protein, alpha subunit precursor	NP_000173
Prohibitin 2 (Phb2)	NP_009204
Solute carrier family 25 (SLC25A5 protein)	AAH68199
	
**Cytoskeleton**	
Actin, gamma 1 propeptide	NP_001605
Adenylyl cyclase-associated protein	NP_006358
Chaperonin containing TCP-1 (subunit 6 A and 7)	NP_006420
Dynein light chain 1	NP_003737
F-actin capping protein alpha 1	NP_006126
Kinesin	NP_004512
Lamin A/C, isoform CRA_c	AAH00511
Lamin B1	NP_005564
Miller-Dieker lissencephaly protein	AAL34972
Myosin heavy polypeptide 9	NP_002464
Myosin, light polypeptide 6B, alkali, smooth muscle and non-muscle, isoform CRA_c	EAW96898
Plectin	NP_958782
t-complex polypeptide 1 (TCP-1)	CAA37064
Tubulin alpha 6	NP_116093
	
**Signal Transduction**	
RAB5C, member RAS oncogene family isoform b	NP_004574
Guanine nucleotide binding protein (G-protein)	EAW53700
IQ Motif containing GTPase activating protein 1	BAA06123
Prohibitin (PHB)	CAG46507
RAN member RAS oncogene familly	EAW98517
	
**Chaperones**	
HSP70-prot 8	NP_006588
HSP60	ABB01006
Tumor rejection antigen (gp96) or Heat schock protein 90 Kda beta	CAI64497
Heat shock protein 90 kDa alpha (HSP90)	NP_005339
	
**Metabolism**	
ACLY variant protein	BAE06117
ATP synthase, H+ transporting, mitochondrial F1 complex, beta subunit precursor	NP_001677
Dihydropyrimidinase-like 2 variant	BAD92432
Enolase 1, variant	BAD96912
Fatty acid synthase	AAA41145
Glucose phosphate isomerase	NP_000166
Glucosidase II	CAA04006
Glyceraldehyde-3-phosphate dehydrogenase	NP_002037
Lactate dehydrogenase A	NP_005557
Peroxiredoxin 6	NP_004896
Phosphoglicerate kinase (PGK)	NP_000282
Phosphoglycerate dehydrogenase	NP_006614
Prostaglandin E synthase 3 (cytosolic)	AAH03005
Pyruvate Kinase, muscle isoform CRA_c	AAH12811
	
**Tumoral Antigen**	
Melanoma-associated antigen 4 (MAGE 4 antigen)	P43358
	
**Calcium binding proteins**	
Annexin 5	NP_001145
Annexine A2 isoform 1	NP_001002858

Proteins from A375 human cells treated with F4 or ethanol (0.2%) as negative control were digested, and the extracted peptides injected onto a 1100 Series HPLC-Chip Cube MS interface, and Agilent 6300 Series Ion Trap Chip-LC-MS/MS system (Agilent Technologies). Data are the proteins down regulated by F4 fraction treatment as judged by mass spectrometry.

**Table 3:** Proteins up regulated by F4 Fraction

**Up regulated Proteins**	**Accesion Number**
**Translational Proteins**	
Alanyl t-RNA synthetase variant	BAD96544
CDA02 (Eukaryotic translation initiation factor 2A)	AAK14926
Eukaryotic translation initiation factor 2, subunit 1 alpha, 35 kDa	NP_001406
Eukaryotic translation initiation factor 3 subunit A, KIAA0139	BAA09488
GA17 protein (eukaryotic translation initiation factor 3, subunit M)	NP_006351
GCN1 general control of amino-acid synthesis 1-like 1, KIAA0219	BAA13209
Leucyl-tRNA synthetase, cytoplasmic, KIAA1352	BAA92590
Methionine adenosyltransferase II, alpha	NP_005902
Mitochondrial isoleucine tRNA synthetase	NP_060530
Prt1 homolog, Eukaryotic translation initiation factor 3 subunit B	AAB42010
Synaptotagmin binding RNA interacting protein, SYNCRIP	AAH24283
	
**Transcription processing**	
DNA-binding protein A (Cold shock domain-containing protein A)	P16989
Heterogeneous nuclear ribonucleoprotein M isoform a	NP_005959
Small nuclear ribonucleoprotein polypeptide F	NP_003086
Small nuclear ribonucleoprotein Sm D1	CAE11897
	
**Ribosomal Proteins**	
Ribosomal protein S15	NP_001004
Ribosomal protein S9	NP_001009
Ribosomal Protein S3A	NP_000996
	
**Protein Degradation**	
Proteasa de Cisteina del Retículo (ER60)	BAA11928
Proteasome 26S ATPase subunit 1 variant	BAD96388
Proteasome 26S ATPase subunit 2	NP_002794
Proteasome 26S non-ATPase subunit 11 variant	BAD96916
Proteasome 26S non-ATPase subunit 2 variant	BAD93080
Putative ubiquitin-conjugating enzyme E2 D3-like protein	Q9NTT1
SUMO1 activating enzyme subunit 1	NP_005491
Tripeptidyl peptidase II	CAH72178
Ubiquitin-Activating enzime E1	NP_003325
	
**Transporters**	
Amino acid transporter E16	AAC61479
ATPase, Ca++ transporting, cardiac muscle, slow twitch 2 isoform 1	NP_733765
Coatomer protein complex subunit alpha isoform 1 (Cop I)	NP_001091868
Exportin 1	NP_003391
Karyopherin beta 1, Importin subunit beta-1	NP_002256
SEC13-like 1 (S. cerevisiae), isoform CRA_b	EAW64078
Signal recognition particle 72 kDa	NP_008878
Solute carrier family 25 (mitochondrial carrier, Aralar), member 12	NP_003696
Stomatin	AAH10703
	
**Cytoskeleton**	
Actin related protein 2/3 complex subunit 2	NP_005722
ARP3 actin-related protein 3 homolog	NP_005712
Chaperonin containing TCP1-subunit 2 beta	EAW97230
Chaperonin containing TCP1-subunit 3 gamma	BAD92119
Destrin, isoform a	NP_006861
Dynactin 1 isoform 1	NP_004073
Dynamin 1-like, isoform CRA_c	EAW88521
Filamin A, FLJ00343	AAF72339
MYO1C variant protein (myosin-I beta)	BAE06097
T-complex protein 1 subunit epsilon, KIAA0098	BAA07894
Transgelin-2, KIAA0120	BAA04802
	
**Cell Cycle**	
Alpha isoform of regulatory subunit A, protein phosphatase 2	NP_055040
Minichromosome maintenance complex component 6	NP_005906
Poly (ADP-ribose) polymerase family, member 1	NP_001609
Regulator of chromosome condensation 1, isoform CRA_c	EAX07692
Septin 9, KIAA0991	BAA76835
	
**Signal Transduction**	
GTP-binding protein PTD004 isoform 1	NP_037473
Phosphofructokinase, platelet, isoform CRA_a	EAW86495
Protein kinase C inhibitor protein 1, YWHAZ	AAH51814
	
**Chaperones**	
Calnexin precursor	NP_001737
Nucleophosmin	AAW67757
Oxygen regulated protein precursor	NP_006380
TNF receptor-associated protein 1 variant	BAD93042
	
**Metabolism**	
5-aminoimidazole-4-carboxamide ribonucleotide formyltransferase	NP_004035
Acyl-CoA synthetase long-chain family 3	NP_976251
Aldehyde dehydrogenase 18 family, member A1	CAI16766
Alkylglycerone phosphate synthase, isoform CRA_b	EAX11058
Carbamoylphosphate synthetase 2/aspartate transcarbamylase/dihydroorotase	NP_004332
Dolichyl-diphosphooligosaccharide-protein glycosyltransferase	CAH73476
Enoyl Coenzyme A hydratase	AAH08906
Glucosamine–fructose-6-phosphate aminotransferase (GFAT 1)	Q06210
HMT1 hnRNP methyltransferase-like 2 isoform 1	NP_001527
Human rab GDI	BAA03095
Hydroxyacyl-Coenzyme A dehydrogenase	AAH14572
Inosine monophosphate dehydrogenase 2, hCG2002013	EAW64946
Ornithine aminotransferase precursor	NP_000265
Phosphogluconate dehydrogenase	NP_002622
Phosphoribosyl pyrophosphate synthetase 2, PRPS2	NP_002756
RPN2	CAG33180
S-adenosylhomocysteine hydrolase	NP_000678

Proteins from A375 human cells treated with F4 or ethanol (0.2%) as negative control were digested, and the extracted peptides injected onto a 1100 Series HPLC-Chip Cube MS interface, and Agilent 6300 Series Ion Trap Chip-LC-MS/MS system (Agilent Technologies). Data are the proteins up regulated by F4 fraction treatment as judged by mass spectrometry.

Efficiency in synthesis of cytoskeleton proteins is required for tumor colony formation, partly explaining why treatment with F4 cells cannot form colonies in soft agar. In addition, these results explain why morphology of treated cells is also abhorrent under light microscope. Also treatment of tumor cell lines with F4 fraction affected proteins associated with metabolism (e.g., peroxiredoxin 6, glucose phosphate isomerase, ACLY variant protein, phosphoglycerate dehydrogenase, pyruvate kinase, muscle isoform CRA, enolase 1, variant Fatty acid synthase, lactate dehydrogenase A, phosphoglicerate kinase (PGK), ATP synthase, H^+ ^transporting, mitochondrial F1 complex, beta subunit precursor, glyceraldehyde-3-phosphate dehydrogenase, glucosidase II, prostaglandin E synthase 3 (cytosolic), dihydropyrimidinase-like 2 variant); some were drastically down-regulated, while others were greatly up-regulated. Chaperone proteins (e.g., Hsp70, Hsp60, tumor rejection antigen (gp96), Hsp90, Hsp90alpha) were also down-regulated after F4 fraction treatment; these proteins are critical for cell survival and protection from stressful stimuli.

## Discussion

Significant attained information from ethnopharmacological reports for our study is the *Petiveria alliacea's *antitumor and immunomodulatory reported activities. To date at a molecular level, there is a lack of scientific evidence to explain such activities. For example, a methanolic extract was unable to induce cytotoxicity on Hep G2 cells. Nonetheless, no specific reason was given for the lack activity in this case [25]. Several compounds isolated from *Petiveria alliacea*, such as astilbin and dibenzyl trisulphide have been demonstrated to induce apoptosis or influence cell cycle or affect actin dynamics [20,26]. The present study demonstrates that *Petiveria alliacea's *F4 fraction contains substances capable of inducing G2 arrest in a dose and time dependent manner (Fig. 5). The ability of F4 fraction to change cell morphology and induce G2 arrest was further investigated. Previous reports demonstrate that dibenzyl trisulphide (DTS), one of the sulfur compounds found in *Petiveria alliacea*, might be responsible for this dual activity [20]. DTS has been previously reported to exhibit potent immunomodulatory function, capable of increasing murine thymic weight along with up-regulation of parameters associated with the reticuloendothelial system, a system essential for molecules involved in immunomodulatory functions [23]. Mice exposed to lethal dose of *E. coli *were protected from death probably because an increase in phagocytic activity [27,28]. DTS has also been reported having anti-fungal activity *in vitro *[16], as well as insecticidal, acaricidal and insect repellent activities *in vivo *[29].

DTS causes reversible microtubule disassembly, which may be due to attenuation of the tyrosyl residues dephosphorylation of the MAP kinases (erk1/erk2) [20]. Along with the fact that MAP kinases are involved in development and apoptotic responses, this event suggests a molecular linkage between these two observations. Mixed-lineage kinase 3 (MLK-3, a kinase of the family controlling MAP kinases activity) inhibition, can cause mitotic arrest by a mechanism involving disruption of microtubule formation and spindle pole assembly [30]. The latter data indicates that *Petiveria alliacea *F4 fraction might inhibit MLK3.

Presence of apoptotic cells after treatment with F4 fraction clearly suggests that cell cycle arrest induces cell death (Fig. 5A). The F4 fraction from *Petiveria alliacea *did not cause mitochondrial membrane depolarization, suggesting that cell death is caused by mitochondrial independent mechanisms (Fig. 4A and 4B). Differentiation of cell death mechanisms, such as necrosis or apoptosis, become necessary since an inflammatory response after tissue injury might be different. The induction of an immune response *in situ *could be the consequence of equilibrium between apoptosis and subsequent necrotic death.

The types of compounds tentatively found in Petiveria alliacea's F4 fraction are sulfur compounds, flavonoids, flavonoid glycosides, coumarin, a monomethylated cyclo hexitol and a fatty acid. The sulfur compounds reported for *Petiveria alliacea *and probably present in F4 fraction are: thiobenzaldehyde S-oxide, dibenzyl sulfide, S-(2-hydroxiethyl)-phenylmetanethiosulfinate, glutamyl-S-benzyl cysteine and dibenzyltrisulfide (Fig. 1B y 1C). It is likely that these compounds are produced by petiverins (benzyl sulfoxides) degradation during the plant extraction process [31], and are associated with antitumor activity. Dibenzyl trisulfide, an immunomodulatory compound isolated from *Petiveria *[20,21], is likely to be present in our fraction. Therefore, could be one of the compounds responsible for the biological activity present in F4 fraction. Pinitol, a monomethylated cyclohexitol reported in *Petiveria alliacea *and possibly present in our fraction, has been reported to exhibit anti-inflammatory properties [32], possibly acting on dendritic cells [33]. Myricitrin, a flavonoid glycoside probably present in F4 fraction, has been reported to have analgesic, anti-inflammatory and antinociceptive properties [34]. Coumarin, another compound possibly found in the F4 fraction is reported to exhibit anti-tumor activity in prostate cancer models [35], and anti-inflammatory activities [36]. Other compounds possibly present in F4 fraction, includes senfol (1,2 diisothiocyanato ethane), 3,5 diphenyltritiolan, 4 ethyl petiveral, 5-O-methyl leridol and lignoceric acid have no literature reports related to anti-tumoral activity.

Down-regulation of cytoskeleton proteins detected by mass spectrometric analysis is consistent with the cytoskeleton disruption observed by fluorescent microscopy. Moreover, changes in the concentration of proteins involved in translation and transduction processes, as well as those involved in cellular metabolism, could explain the decrease of tumor cells clonogenic ability, as well as the anti-tumor activity of *Petiveria alliacea*. Currently, we are evaluating the coding genes for these proteins in order to determine if the changes are at the transcriptional level or whether the proteomic results are a consequence of differential management of the existing proteins in the tumor cells. The mechanism by which tumor cells undergo death should be determined. Our results indicate that there is DNA fragmentation; however, it is possible that oxidative stress, metabolic changes, necrosis or senescence are also ways by which tumor cells may undergo death. In fact, necrotic death can provide the necessary danger signals to induce dendritic cells activation, giving anti-tumoral protective immune response [37]; although other mechanisms can be implied in this antigen transfer [38,39]. Induction of an effective immune response is unknown, but possibly *Petiveria alliacea *F4 fraction, can act as Sho-Saiko-to, or Juzen-taiho-to [40,41], inducing reduction of primary tumors, metastasis, and generating a specific CD8+ CTL responses. Mechanisms implied in the process are unknown. However, it is critical to understand and elucidate the molecular mechanisms before the plant fraction can be used in the design of effective cancer drug therapeutics.

## Conclusion

In conclusion, our study demonstrates that *Petiveria alliacea's *F4 fraction, exhibits multiple anti-tumoral activities against human (K562, A375) and mouse (Mel Rel) tumor cells. F4 fraction exerts G2 cell cycle arrest, induces actin cytoskeleton reorganization, affects cell morphology, causes DNA fragmentation and decreases clonogenicity. Furthermore, our findings indicate that F4 fraction may use multiple molecular targets to exert its antitumor activity.

## Abbreviations used

EtOH: ethanol; EtOAc: ethyl acetate; Hsp70: seventy kilo-Dalton heat shock protein; MeOH: methanol; MLK-3: mixed-lineage kinase 3; MTT: 3-(4,5-dimethylthiazol-2-yl)-2,5-diphenyltetrazolium bromide; PBMC: peripheral blood mononuclear cells; PAF: paraformaldehyde; PBS: phosphate buffer saline.

## Competing interests

The authors declare that they have no competing interests.

## Authors' contributions

The present work was conceived, directed and coordinated by SF helped by AA. Biological assays, cell line maintenance, viability tests, cell cycle and cytoskeleton analysis, DAPI DNA fragmentation test and protein expression analysis by CU. CC, performed the preparation and characterization of the plant extracts by de-replicacion, DC, performed mitochondrial membrane depolarization tests and AA, performed clonogenicity tests. PK, performed protein LC-MS/MS sample preparation and analysis by Spectra Mill bioinformatics software. All authors have read the manuscript and agree to its contents.

## Pre-publication history

The pre-publication history for this paper can be accessed here:


